# Barriers and Facilitators to Physician-Patient Communication in Chinese Tertiary Hospitals From the Perspectives of Hospital-Based Physicians and Patient Relations Coordinators: Qualitative Study

**DOI:** 10.2196/87947

**Published:** 2026-06-11

**Authors:** Zengping Shi, Qinqin Jiang, Xincheng Wang, Hongli Yan, Yi Xia, Lushaobo Shi, Dong Wang

**Affiliations:** 1 School of Public Health, Southern Medical University, No.1023 Shatai Road, Guangzhou, Guangdong, 510515, China, 86 02061648077; 2 School of Health Management, Southern Medical University, Guangzhou, Guangdong, China; 3 The Public Health Policy Research and Evaluation Key Laboratory Project of the Philosophy and Social Sciences of Guangdong College, Southern Medical University, Guangzhou, Guangdong, China; 4 Southern Medical University Center for Health Policy and Governance (Guangdong Provincial Social Science Research Base), Southern Medical University, Guangzhou, Guangdong, China; 5 Research Base for Development of Public Health Service System of Guangzhou, Southern Medical University, Guangzhou, Guangdong, China; 6 Center for Faculty Development and Research, Guangzhou Medical University, Guangzhou, Guangdong, China

**Keywords:** physician-patient communication, communication barriers, communication facilitators, qualitative research, tertiary hospitals

## Abstract

**Background:**

Effective physician-patient communication is essential for building trust and sustaining positive relationships, yet becomes increasingly challenging in China’s tertiary hospitals, where physicians face heavy workloads.

**Objective:**

This study explored the barriers and facilitators of physician-patient communication by synthesizing perspectives from Chinese hospital-based physicians and patient relations coordinators.

**Methods:**

A qualitative study using semistructured interviews was conducted with 17 participants (11 physicians and 6 patient relations coordinators) from tertiary hospitals in China (April-July 2025). Data were analyzed using thematic analysis following Braun and Clarke’s framework.

**Results:**

Barriers and facilitators of physician-patient communication were organized into a multilevel framework comprising patient-level, physician-level, and system-level factors. Patient-level barriers included individual background differences, inadequate expression and limited health literacy, and psycho-emotional states with expectation misalignment, whereas facilitators included effective expectation management, good health literacy and communication readiness, and trust in physicians with shared decision-making. Physician-level barriers involved communication style deficiencies, empathy gaps, and time pressure constraints; facilitators included active listening and patient-centeredness, empathy and emotional support, and clear explanations with cultural adaptability. At the system level, barriers included hospital environment and medical visit settings, legal and policy deficiencies, insufficient communication training, and media-driven distrust with digitally mediated information challenges, while facilitators included institutional support and security assurance, educational training and policy promotion, process optimization and patient health education, and artificial intelligence–assisted baseline knowledge.

**Conclusions:**

Physician-patient communication is influenced by multiple factors, necessitating comprehensive intervention measures: enhancing patient education, improving physician communication skills, and strengthening organizational support systems. Notably, special attention should be directed toward addressing the unique challenges posed by digital technologies while concurrently leveraging the opportunities they present to optimize communication outcomes.

## Introduction

As global health care systems increasingly contend with the challenges of aging populations, constrained resources, and growing patient expectations, effective physician-patient communication has become more crucial than ever, serving as a cornerstone of high-quality health care delivery [1,2]. A growing body of evidence shows that high-quality communication enhances patient satisfaction, improves treatment adherence, reduces medical errors, and ultimately contributes to better health outcomes [3-5]. Conversely, communication failures can have serious consequences; for instance, Humphrey et al [6] reported that an analysis of malpractice claims revealed that 49% involved communication failures and 53% specifically reflected physician-patient miscommunication.

Effective physician-patient communication is inherently a 2-way interaction in which both physicians and patients seek to understand and be understood [7-9]. However, current research on improving physician-patient communication has focused mainly on meeting patients’ needs, with particular emphasis on improving physicians’ communication skills [10-12]. This patient-centered approach has prioritized patient-reported experiences and satisfaction measures [13] while largely neglecting physicians’ reciprocal need to be understood and their perspectives on physician-patient communication challenges.

Moreover, previous studies on physician-patient communication have mostly focused on Western family medical settings, with limited attention to the Chinese context. Yet the determinants of physician-patient communication vary significantly across cultural contexts [14]. Especially within China’s inverted triangle health care system [15], tertiary hospitals serve as regional medical centers characterized by high patient volumes, short consultation times, and cross-regional patient mobility. Compared to secondary and primary hospitals, physicians at tertiary hospitals must balance heavy clinical workloads with patients’ expectations for detailed explanations [16]. At the same time, the digital transformation of health care, including increased access to online health information, platform-based services, and emerging technologies such as artificial intelligence (AI), is reshaping the context in which physician-patient communication takes place. While these developments may create new opportunities for information exchange, they may also intensify misunderstandings, alter expectations, and introduce new communication challenges that remain insufficiently understood [17,18]. As frontline practitioners experiencing the dual pressures of system strain and digital transformation, physicians in tertiary hospitals may offer valuable insights into these evolving communication challenges. In addition, patient relations coordinators, who are directly involved in managing complaints and mediating disputes, can provide important perspectives on how these challenges manifest in practice and where systemic gaps may exacerbate tensions.

This study explored barriers and facilitators to physician-patient communication from the perspectives of frontline physicians and patient relations coordinators in Chinese tertiary hospitals. By integrating a dual-role perspective, this study aimed to develop a more nuanced understanding of the complex factors shaping physician-patient communication in contemporary hospital settings.

## Methods

### Study Design and Participants

We conducted a qualitative descriptive study using individual in-depth interviews to explore the perspectives of physicians and patient relations coordinators in Chinese tertiary hospitals regarding physician-patient communication. This study was situated within a critical realist ontological framework, which acknowledges that participants’ experiences reflect real phenomena in clinical settings, while recognizing that our understanding of these phenomena is inevitably mediated by individual perspectives, social contexts, and the interpretive lens of the research team [19]. The study followed the COREQ (Consolidated Criteria for Reporting Qualitative Research) checklist [20] and was conducted from April to July 2025 in Guangdong Province, China.

Participants were recruited from multiple tertiary hospitals through purposive sampling. Recruitment was initiated by the first authors through direct solicitation, under the guidance of the corresponding author, and was subsequently expanded during data collection to ensure diversity in institutional contexts and participant characteristics. The sampling strategy aimed to achieve variation in professional roles, gender, age, years of experience, and clinical specialty, thereby capturing a broad range of perspectives on physician-patient communication.

A total of 17 participants were recruited from tertiary hospitals, including 11 physicians from 7 tertiary hospitals and 6 patient relations coordinators from 5 tertiary hospitals, representing 11 tertiary hospitals in total across the full sample. The distribution of participants across institutions is detailed in Table 1. These 2 participant groups were purposively selected because they offered distinct yet complementary perspectives on physician-patient communication. Practicing physicians across different clinical departments were recruited to capture firsthand physician-patient communication experiences. Patient relations coordinators were included because of their roles in handling complaints, coordinating communication, mediating disputes, and identifying institutional barriers affecting physician-patient communication. Including both groups enabled the study to integrate direct clinical perspectives with insights from the hospital organizational level. All participants were currently employed, fluent in Mandarin, and had a minimum of 1 year of professional experience to ensure sufficient exposure to physician-patient communication dynamics. Retired practitioners were excluded from the study to ensure perspectives reflected current practice contexts.

**Table 1.** Sample characteristics (N=17).

ID	Gender	Age (y)	Years of service	Hospital ID	Department	Education level	Professional title	Administrative position
Physicians (n=11)								
Dr01	Male	46	17	H1	General Medicine	Master	Associate Senior	Deputy Director of Department
Dr02	Male	48	26	H1	Respiratory Medicine	Bachelor	Senior	Deputy Director of Department
Dr03	Female	35	10	H2	General Medicine	Bachelor	Junior	None
Dr04	Female	46	14	H3	Pulmonary Function Laboratory	Doctor	Associate Senior	None
Dr05	Male	29	3	H4	Ophthalmology	Bachelor	Junior	None
Dr06	Male	42	15	H5	Traditional Chinese Medicine (Ancient Formulas)	Master	Intermediate	None
Dr07	Male	50	30	H5	Cardiology	Doctor	Associate Senior	Director of Department
Dr08	Male	47	22	H6	General Medicine	Bachelor	Intermediate	None
Dr09	Male	38	10	H6	Cardiology	Master	Intermediate	None
Dr10	Male	38	10	H7	Spinal Surgery	Doctor	Associate Senior	None
Dr11	Female	35	10	H7	Obstetrics and Gynecology	Bachelor	Intermediate	None
Patient relations coordinators (n=6)								
A01	Female	31	8	H8	Physician-Patient Relations Office	Bachelor	None	None
A02	Male	37	16	H9	Physician-Patient Relations Office	Bachelor	Intermediate	Deputy Director of Department
A03	Female	39	3	H10	Physician-Patient Relations Office	Bachelor	None	None
A04	Female	33	8	H6	Physician-Patient Relations Office	Master	None	None
A05	Female	37	16	H6	Physician-Patient Relations Office	Bachelor	Junior	None
A06	Female	27	1	H11	Physician-Patient Relations Office	Master	None	None

Recruitment and data collection proceeded iteratively. The study sample size was informed by the concept of information power [21], which considers the study aim, sample specificity, theoretical framework, quality of dialogue, and analytic strategy. Given the focused aim of the study, the use of reflexive thematic analysis, and a purposively diverse sample drawn from 2 relevant participant groups with direct experience of physician-patient communication processes, we anticipated that a relatively modest sample would yield sufficient depth. Throughout data collection, we monitored the richness and relevance of interview material in relation to the study aims. We concluded recruitment at 17 participants when the research team collectively assessed that the interviews had generated sufficiently rich and diverse data to develop coherent analytic themes addressing the research question, and that additional interviews were unlikely to substantially alter the developing analysis.

### Data Collection and Procedures

Data were collected through semistructured, one-to-one interviews. Participants who could not meet in person were interviewed online via the Tencent Meeting platform, while others participated in face-to-face interviews. All interviews were conducted in Mandarin, lasted 45‐90 minutes, and were led primarily by ZS, with HY moderating 3 interviews. Demographic data were collected verbally at the beginning of each interview. All interviews were audio- or video-recorded with participants’ consent, transcribed verbatim by professional transcriptionists within 24 hours of completion to preserve contextual detail and support timely familiarization with the data. A random sample of 20% of transcripts was independently verified against original recordings by research team members to ensure transcription accuracy. Participant quotes selected for inclusion were translated into English by one bilingual research team member (ZS). To ensure conceptual equivalence and linguistic accuracy, a second independent bilingual team member (QJ) who had no prior exposure to the original Chinese text backtranslated all quotes into Mandarin. Discrepancies between the original and backtranslated versions were reviewed and resolved through consensus discussion among the research team until conceptual equivalence was confirmed. Participants received RMB 150 (approximately US $20) as compensation for their time.

A semistructured interview guide was developed collaboratively by the research team (Multimedia Appendix 1) and refined iteratively based on insights from initial interviews. The interview guide explored four core domains: (1) key physician behaviors and patient preparations that support high-quality interactions, (2) patients’ main needs and expectations, (3) characteristics of effective patient-physician communication from the physician’s perspective, and (4) personal, situational, and systemic barriers and facilitators to effective physician-patient communication. Throughout the interviews, the research team used techniques such as probing questions, clarification requests, and reflective listening [22] to elicit in-depth responses and encourage participants to share nuanced insights about the facilitators and barriers to physician-patient communication.

To ensure the scientific rigor and quality of data collection, several measures were implemented. All interviewers (ZS and HY) underwent training in qualitative interview techniques and conducted mock interviews prior to data collection to standardize approaches and ensure consistency. All interviews were conducted in quiet, private settings with standardized protocols, including consistent opening statements and informed consent procedures. For online interviews, technical equipment was tested beforehand to ensure optimal audio and video quality. Interviewers maintained brief reflective notes during and immediately after each interview to capture contextual observations, nonverbal cues where available, and preliminary reflexive insights.

### Researcher Reflexivity Statement

Given the interpretive nature of reflexive thematic analysis, it is important to acknowledge the positionality and perspectives that the research team brought to this study [23,24]. The lead researcher and primary interviewer (ZS) is a doctoral researcher with training in qualitative research methods and prior professional experience in culture- and communication-related work at a tertiary hospital in China. This background provided familiarity with the dynamics of physician-patient communication in China’s health care system, which facilitated rapport-building with participants and sensitivity to contextual nuances during interviews. At the same time, this proximity required reflexive awareness of potential assumptions about what constitutes “effective” communication, and ZS took care to remain open to perspectives that might differ from her own professional experiences.

The broader research team comprised members with expertise in clinical medicine, health communication, qualitative methodology, and health services research, which enriched the analytic process by bringing multiple interpretive lenses to the data. ZS maintained brief reflexive notes after each interview and met regularly with the supervisory team to discuss emerging interpretations, challenge assumptions, and consider how the team’s positionality may have shaped the developing analysis. These reflexive practices were integral to the analytic process and supported the generation of themes that were grounded in participants’ accounts while acknowledging the research team’s active role in interpretation [19].

### Data Analysis

Interview transcripts were analyzed using reflexive thematic analysis following Braun and Clarke’s 6-step framework [23-25], which emphasizes the researcher’s active and reflexive role in generating themes through deep engagement with the data. An inductive, data-driven orientation was adopted, whereby codes and themes were developed in response to participants’ accounts without the imposition of a predetermined framework. Data management and coding were conducted using NVivo 20 (Lumivero LLC). Participant quotes were coded using alphanumeric identifiers: “Dr” followed by numbers (Dr01-Dr11) for practicing physicians, and “A” followed by numbers (A01-A06) for members from physician-patient relations management departments.

ZS led the analysis, conducting inductive coding of all transcripts through an iterative process of close reading, annotation, and code development. To deepen analytic reflexivity and broaden interpretive engagement with the data, XW independently coded a subset of transcripts (the first 2 transcripts from each participant group). These additional coding contributions informed collaborative discussions about alternative readings and emerging patterns, contributing to the refinement of the developing analytic framework. The purpose of this collaborative coding was not to achieve consensus or establish interrater agreement, but rather to bring multiple interpretive perspectives to the data and to deepen the team’s reflexive engagement with the analysis.

The team met regularly during the analytic process to discuss coding decisions, review evolving themes, and critically examine the developing analysis. DW provided an additional interpretive perspective during theme refinement. The final thematic framework was developed through an iterative process of deep engagement with the data across all phases of analysis, moving between data, codes, and candidate themes in a recursive manner. Themes were generated as interpretive patterns of shared meaning across the dataset, rather than predetermined categories [23]. In keeping with reflexive thematic analysis, analytic rigor was supported through sustained engagement with the full dataset, reflexive memoing, and regular interpretive discussions within the research team, rather than through coder agreement or the resolution of coding discrepancies to achieve consensus.

### Trustworthiness and Quality

Several strategies were used to enhance the trustworthiness of the study, guided by considerations of credibility, transferability, dependability, and confirmability [26], while remaining attentive to the reflexive and interpretive nature of the analytic approach [23].

Credibility was supported through prolonged engagement with the data, purposive sampling to capture diverse perspectives across clinical specialties and professional roles, and the inclusion of 2 complementary participant groups (physicians and patient relations coordinators) to provide a multifaceted understanding of physician-patient communication. Collaborative coding of a subset of transcripts and regular team discussions further enriched the analytic process by introducing multiple interpretive perspectives.

Transferability was enhanced through a detailed description of the study context, participant characteristics, and analytic procedures, enabling readers to assess the applicability of the findings to their own settings. The purposive sampling strategy ensured diversity in institutional contexts, clinical specialties, and professional roles across the sampled tertiary hospitals in Guangzhou and Shenzhen, Guangdong Province, China.

Dependability was addressed through transparent documentation of all research decisions throughout the study. The interview guide, coding process, and thematic development were documented and discussed within the research team, creating an audit trail that traces the analytic journey from raw data to final themes.

Confirmability was supported through reflexive practices, including the maintenance of reflexive notes by interviewers, regular supervisory discussions about emerging interpretations, and the explicit acknowledgment of the research team’s positionality and its potential influence on the analysis. Within the framework of reflexive thematic analysis, we recognize that analysis is inherently shaped by the researcher’s subjectivity; therefore, confirmability was pursued not through the elimination of researcher influence, but through transparency about the interpretive process [19,27].

### Ethical Considerations

This study was approved by the Ethics Committee of Southern Medical University, Guangzhou, Guangdong Province, China (approval number NFYKDX003-2025-35), and was conducted in accordance with the Declaration of Helsinki. Prior to the interviews, participants were informed about the purpose of the study and the use of audio recording, and informed consent was obtained from all participants. Interviews were audio-recorded only with participants’ permission. No personally identifiable information was collected or reported, and all data were kept confidential.

## Results

### Demographics of the Participants

We interviewed a total of 17 participants, comprising 11 physicians and 6 patient relations coordinators (Table 1). Among the physicians, there were 8 males and 3 females, with a mean age of 41.27 (SD 6.40; range 29‐50) years and a mean professional experience of 15.18 (SD 7.65; range 3‐30) years. They represented a broad range of specialties, including obstetrics and gynecology, cardiology, spinal surgery, general medicine, and other specialties. Given China’s high patient-to-physician ratio and the central role of tertiary hospitals in delivering specialist care, physicians with senior roles (eg, department directors or deputy directors) remain active frontline clinicians regardless of administrative responsibilities. Three physicians with administrative roles reported conducting outpatient consultations on a regular basis, averaging 3 to 4 sessions per week. Among the patient relations coordinators, there were 1 male and 5 females, with a mean age of 34.00 (SD 4.12; range 27‐39) years and a mean professional experience of 8.67 (SD 5.61; range 1‐16) years.

### Overview of Themes

**Figure 1.**
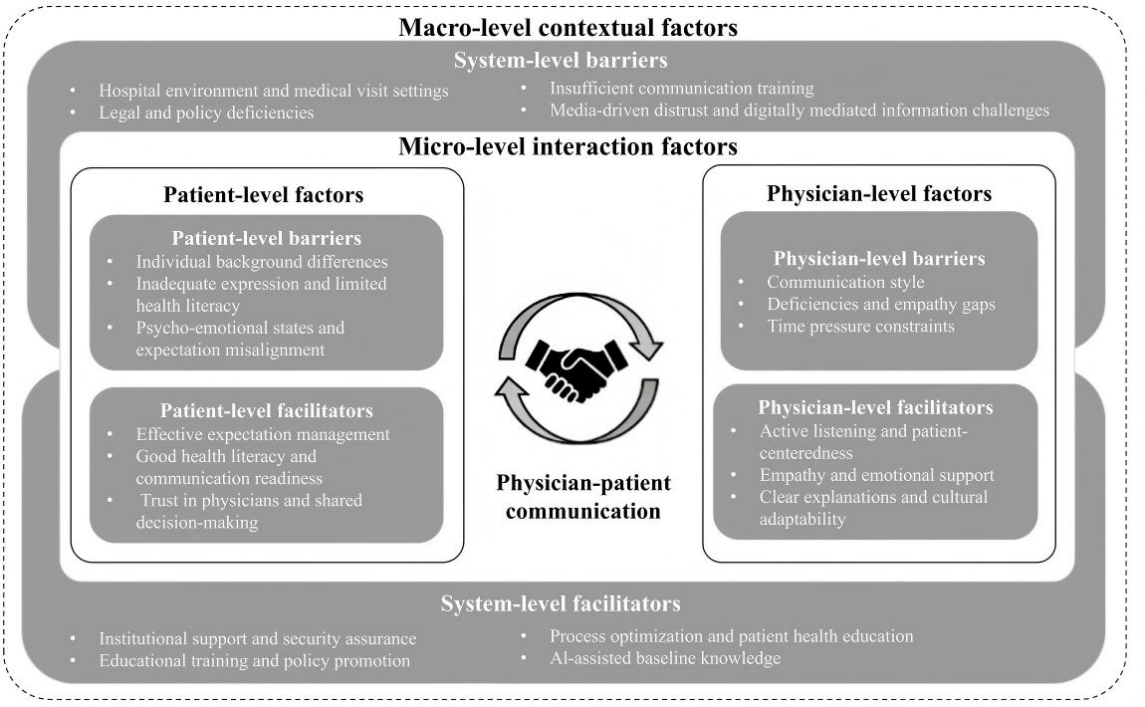
Overview of the themes. AI: artificial intelligence.

Thematic analysis of the interview data identified 2 overarching themes: barriers and facilitators of effective physician-patient communication. As shown in Figure 1, these themes were organized into a multilevel framework comprising patient-level, physician-level, and system-level factors. Patient- and physician-level factors directly shaped the communication process, whereas system-level factors functioned as the broader context that could either hinder or enable effective interaction. The following sections discuss each theme and its constituent subthemes in detail.

### Theme 1: Barriers to Effective Physician-Patient Communication

#### Patient-Level Barriers

##### Individual Background Differences

Physicians reported that patients’ educational levels directly influenced communication effectiveness, with particularly notable differences between highly educated and less educated patients. Many highly educated patients tended to arrive with strong preformed views about their conditions, sometimes displaying overconfidence and questioning physicians’ clinical judgments. Conversely, patients with limited education required extensive, repeated explanations to understand medical information, frequently resulting in miscommunication. For these patient groups, the standardized communication methods taught in medical schools were largely ineffective, and time constraints in high-volume outpatient settings made it difficult to tailor communication strategies to individual patients. As one physician explained,

For some well-educated patients, the problem lies in overconfidence; for others, I need to explain and rephrase multiple times before they can understand what I mean.

[Dr05]

Patient age also shaped communication dynamics. Younger patients demonstrated higher autonomous learning motivation and were more likely to form preliminary self-diagnoses before consultations, a tendency exacerbated by the digital information environment elaborated in the subsequent section (see “System-Level Barriers”). Furthermore, they often approached health care from a consumerist perspective, expecting guaranteed outcomes and engaging in what participants described as excessive rights protection behaviors, such as recording consultations and filing complaints. Such behaviors often prompted physicians to adopt defensive communication practices, including avoiding discussions of clinical uncertainty. As a member of the physician-patient relations office noted,

Many young patients treat medical consultations like ‘online shopping,’ expecting guaranteed treatment outcomes and a ‘seven-day free return’ policy. However, medicine is inherently fraught with uncertainty, and no one can guarantee outcomes...Some patients record every interaction, forcing physicians to speak with excessive caution and hesitate to discuss treatment uncertainties.

[A03]

##### Inadequate Expression and Limited Health Literacy

Inadequate symptom expression and limited health literacy are the primary patient-level barriers to physician-patient communication. Physicians reported that many patients lacked the ability to accurately describe symptoms and express their core consultation needs clearly. Patients often used colloquial language, requiring physicians to adjust their questioning strategies repeatedly and clarify complaints through follow-up inquiries. Even so, patients frequently provided irrelevant responses, resulting in the loss of key clinical information. One physician elaborated on the difficulty of obtaining effective clinical information:

When I ask under what specific circumstances the stomach pain occurs, they just repeat ‘severe bloating’ without answering the actual question...Sometimes we spend five minutes and still cannot obtain the necessary information, which leaves us feeling quite helpless.

[Dr07]

One physician further noted,

Patients who can clearly express their conditions and are quite professional are actually rare... The health literacy of most patients in China still has significant room for improvement.

[Dr01]

Beyond incomplete symptom descriptions, participants emphasized a significant disconnect between patients’ surface-level expressions and their underlying needs. One physician added,

Some patients are unable to clearly articulate their true needs; in fact, what a patient says during a consultation may not reflect their actual needs. If the doctor cannot discern this, communication will still fail even if the diagnosis is correct.

[Dr05]

##### Psycho-Emotional States and Expectation Misalignment

Physicians described encountering contradictory patient demands and misaligned expectations during treatment processes. On one hand, patients’ psycho-emotional status, such as disease-related anxiety, significantly disrupted communication. One physician provided a vivid example:

A patient might just have a sore throat, but it is accompanied by overwhelming anxiety, fearing they would be reprimanded by their boss, and might even lose their job.

[Dr03]

Such intense emotional states caused patients to talk excessively and pose repetitive questions, overwhelming the physician and impeding effective clinical communication. On the other hand, expectation misalignment was also evident when patients sought definitive diagnoses or guaranteed outcomes while resisting the examinations or costs required to support clinical decision-making. As one physician stated,

Some patients hope for both definitive diagnosis and cost savings, which is somewhat contradictory in certain situations. For example, to give a definitive diagnosis, I need to order some examinations to find the root cause. Naturally, this requires more examinations, but once examinations increase, patients feel that costs might be higher, so contradictions exist here.

[Dr01]

### Physician-Level Barriers

#### Communication Style Deficiencies and Empathy Gaps

Physicians’ professional capabilities directly influenced their ability to understand and manage patient conditions. Good professional competence helped enhance patient trust and treatment compliance; conversely, insufficient professional capabilities not only weakened therapeutic effects but also reduced communication effectiveness. As one physician noted,

If I cannot clearly explain or show confidence in diagnosis, patients will naturally doubt what I say.

[Dr02]

Some physicians exhibited authoritative tendencies in communication, often assuming patients could understand medical concepts while lacking the ability to translate professional information into accessible language. Communication involved excessive use of medical terminology and rigid language, tending toward unidirectional information delivery rather than shared decision-making with patients, sometimes displaying condescending attitudes. As a member of the physician-patient relations office observed,

Many physicians tend to dominate conversations. They use many professional terms, but patients often have no idea what they mean.

[A06]

This approach not only weakened patient participation but also exacerbated power imbalances between physicians and patients, making patients feel ignored or disrespected. Furthermore, when facing physician-patient conflicts, some physicians lacked awareness of appropriate vulnerability demonstration and empathy expression, requiring improvement in communication skills. A physician remarked,

Physician-patient conflicts often escalate from verbal confrontations. In some cases, some conflict escalations occur when physicians insist on verbal dominance and want to win the argument. When patients feel disadvantaged in communication process, they may turn to other forms of confrontation, such as calling 12,315 (Chinese consumer rights complaint hotline), or engaging in physical confrontation. In my view, physicians do not need to compete for victory; instead, they can show appropriate vulnerability and defuse conflicts before they escalate beyond verbal confrontation.

[Dr02]

#### Time Pressure Constraints

Insufficient time was the most frequently mentioned communication barrier by physicians. Participants’ accounts showed that high patient volume, limited staffing, and heavy workload reduced the time physicians could allocate to each consultation. Several physicians described that, although they valued communication and recognized its importance in clinical care, time pressure often constrained them to provide detailed condition explanations. For instance, one physician stated,

Patients might wonder why physicians can’t give them more time. I also want to give them plenty of time, but I need to see 50 patients in one morning, with over ten people still waiting. My work pressure is also enormous; I can only try my best to do well.

[Dr05]

Likewise, one physician noted,

it’s almost time to clock out, and there are still 10 to 20 people are queuing behind you...objectively, there is simply no time.

[Dr09]

This perception was also echoed by a member of the physician-patient relations office, who commented,

Some physicians see 50 patients in a single morning. Do they realistically have the time to explain everything to each patient individually? Not possible.

[A06]

### System-Level Barriers

#### Hospital Environment and Medical Visit Settings

Any problems in the medical care-seeking process, such as long waiting times or cumbersome examinations leading to poor visit experiences, could cause patients to project emotions onto physicians with whom they directly interacted. Emotional venting was one of the important causes of physician-patient communication conflicts. Some patients carried emotions from negative previsit experiences into communications with physicians. A member of the physician-patient relations office described,

The tipping point for a patient’s emotional outburst is rarely an isolated incident; rather, it is the culmination of their preceding experiences. For example, patients waited one or two hours to see the physician, the consultation is over in just two or three minutes. After that, patients have to go through examinations, wait for reports, make payments, and return visits. The whole process is very exhausting.

[A02]

#### Insufficient Communication Training

Although medical education invested heavily in clinical skill development, physician-patient communication training remained relatively underdeveloped, with curriculum content primarily focused on diagnosis and technical operations, while communication training for high-complexity clinical scenarios and patient emotional and psychological needs remained insufficient. This problem was particularly prominent among young physicians. Limited by clinical experience, they often lacked confidence in communication and struggled to quickly establish trust; simultaneously, patients questioned the professional authority of junior physicians, further exacerbating communication barriers. A member of the physician-patient relations office noted,

In school, physician-patient communication was not a core curriculum. Although hospitals provide training after employment, genuine communication skill improvement still depends on personal understanding. Because communication scenarios are complex, merely teaching communication theory cannot actually solve practical problems. Communication is a capability requiring lifelong learning.

[A04]

#### Legal and Policy Deficiencies

Insufficient legal enforcement and protection mechanisms left both patients and physicians feeling vulnerable. Physicians reported that patients currently possessed numerous accessible channels for expressing demands; however, physicians lacked adequate advocacy channels when facing unreasonable demands, verbal abuse, or violence. This “legal vacuum” forced medical staff to adopt defensive postures, hindering honest dialogue about medical uncertainty or treatment limitations. Additionally, complex medical insurance policies (such as reimbursement scope and self-payment ratios) often became “noise” in physician-patient communication. Prescription authority restrictions, medical insurance reimbursement clauses, or hospital regulations often forced physicians to refuse patient requests. This “institutional rejection” was easily misunderstood by patients as physician indifference, exacerbating relationship tensions. As one physician stated,

If I cannot be certain of receiving protection when problems arise, I will avoid discussing any potentially controversial content.

[Dr11]

One physician expressed similar difficulties,

Sometimes I must say ‘no’ due to regulations, but patients think I simply don’t care.

[Dr04]

#### Media-Driven Distrust and Digitally Mediated Information Challenges

Participants described media narratives and digital health information environments as important barriers to effective physician-patient communication. For one thing, biased media coverage of negative medical incidents was found to foster patients’ preconceived distrust of physicians prior to consultations, which directly hindered the establishment of therapeutic rapport, mutual trust, and open communication. As one physician observed,

Some media’s negative medical reporting affects patients’ impressions of physicians. Patients believe physicians are making money from patients’ illnesses, arriving with such preconceived notions before visits. In such situations, it’s very difficult for physicians to change these prejudices through communication, so public opinion direction is extremely important.

[Dr07]

On the other hand, the growing use of digital technologies and the wide availability of online health information introduced new communication challenges. Physicians reported that patients increasingly searched for health information online and, in some cases, used AI tools such as DeepSeek (Hangzhou DeepSeek Artificial Intelligence Co, Ltd) before or after consultations. Patients were often described as becoming partially informed through fragmented online or AI-generated information, without fully understanding the broader clinical context. This increased the explanatory burden on physicians and sometimes shifted consultations away from treatment planning toward the correction of misinformation. As one physician explained,

More and more patients come with DeepSeek recommendations. Although I’m willing to maintain an open attitude to communication, each time I need to spend time mutually sharing materials and judging which side is more reasonable and has stronger evidence, it’s quite exhausting. Sometimes I spend more time explaining why AI recommendations aren’t suitable for patients than discussing actual treatment plans.

[Dr09]

### Theme 2: Facilitators of Effective Physician-Patient Communication

#### Patient-Level Facilitators

##### Effective Expectation Management

Effective management of expectations was identified as a key characteristic of patients that facilitates constructive physician-patient communication. Participating physicians clearly preferred patients who understood the limitations of medicine, the complexities of diagnosis, and the uncertainties of treatment outcomes. As a member of the physician-patient relations office explained,

Many patients expect health to be a completely disease-free state. But medicine has its limitations. Physicians can only promise to improve conditions through treatment; a complete absence of disease is impossible. If patients can manage their expectations, we can have peaceful discussions about what is possible, rather than being forced to make promises that we cannot fulfill.

[A01]

Physicians noted that such patients enabled more open and honest discussions about treatment options, potential outcomes, and associated risks.

##### Good Health Literacy and Communication Readiness

Patients’ strong communication skills and adequate health literacy helped improve physician-patient interaction quality. Patients who could clearly present medical histories, ask targeted questions, and demonstrate understanding of basic medical concepts enabled physicians to focus more on complex decisions and personalized treatment.

If patients can clearly describe symptoms and understand basic terminology, we can spend more time developing treatment plans rather than starting from scratch.

[Dr11]

Patients’ strong sense of health responsibility was also emphasized as a valuable factor promoting physician-patient communication. Physicians valued well-prepared patients who maintained organized medical records, obtained information from reliable sources, and asked clear questions.

A patient who communicates well knows they are the primary person responsible for their own health, rather than believing that medical visits are entirely the physician’s responsibility. Patients can prepare their past medical history materials as much as possible, allowing us to communicate and interact, examine disease development trends, making the entire diagnostic and treatment process smoother and more efficient.

[Dr09]

##### Trust in Physicians and Shared Decision-Making

Patient trust in physicians and shared decision-making more easily helped patients and physicians establish balanced and mutually trusting dialogue atmospheres. Physicians emphasized preferring patients who respected their professional knowledge while asking insightful questions, with shared decision-making during communication interactions being more aligned with patient needs.

Trust is the foundation. When patients trust physicians but still ask questions, this means they are actively participating in communication. This type of communication interaction more easily facilitates shared decision-making, helping patients choose treatment plans most suitable for their current personal situations.

[Dr01]

### Physician-Level Facilitators

#### Active Listening and Patient-Centeredness

Participating physicians emphasized that active listening and patient-centeredness were foundations of effective medical communication. Through attentive listening, asking follow-up questions based on patients’ verbal or nonverbal cues, and summarizing their core concerns, physicians not only made patients feel heard and valued but also reduced risks of missing important symptoms or worries. One physician mentioned,

I believe treating illness requires standing from the patient’s perspective, first treating the heart. Many patients don’t have physical illness but anxiety. When physicians patiently listen to their complete expressions, patients may feel half-better upon leaving the consultation room.

[Dr10]

#### Empathy and Emotional Support

Empathy and emotional support were repeatedly mentioned as core elements for building trust and enhancing treatment compliance. Physicians who could recognize patient fears, acknowledge their concerns, and provide comfort could establish relationships transcending clinical interventions. Such connections were viewed as key to alleviating anxiety and enhancing patient confidence in treatment processes.

I believe a very important aspect of being a doctor is having understanding and empathy, being able to comprehend why patients say what they do; additionally, being able to make patients feel ‘this physician understands me’ from the first words spoken.

[Dr05]

#### Clear Explanations and Cultural Adaptability

Using accessible language for clear explanations was viewed as key to bridging gaps between medical expertise and patient understanding. Physicians described the importance of translating professional terminology into colloquial language, using analogies or visual aids when appropriate, and confirming patient comprehension levels to improve communication. One physician emphasized,

Many medical professional terms are incomprehensible to patients; we need to translate professional terms into common people’s language, making them understandable and clear. [Dr02]

Maintaining transparency when discussing diagnoses, treatment options, and prognoses was also viewed as a hallmark of effective communication. Physicians who frankly discussed uncertainties, limitations, and potential complications could maintain hope while setting realistic expectations, thereby avoiding disappointment and conflict. Many associated this transparency with more collaborative relationships, especially when combined with shared decision-making approaches that incorporated patient preferences and values.

I told them frankly what we knew, what we didn’t know, and what the risks were, and then we decided together how to proceed.

[Dr11]

When interacting with diverse patient populations, cultural sensitivity and adaptive communication styles were viewed as crucial. Recognizing differences in health beliefs, communication preferences, and decision-making processes enabled physicians to adjust communication approaches accordingly, reducing misunderstandings and promoting respectful, culturally appropriate care. As one physician explained,

If you don’t understand the patient’s cultural background, you may be talking, but you’re not really connecting.

[Dr11]

### System-Level Facilitators

#### Institutional Support and Security Assurance

Physicians unanimously emphasized that institutional support and security assurance provided by medical institutions were important prerequisites for physicians to conduct effective communication. For example, hospitals’ zero-tolerance policies toward violence and abuse, along with clear behavioral standards and response mechanisms, made physicians feel more secure and confident when communicating with patients, thereby reducing defensive behaviors and promoting open dialogue. Transparent and efficient complaint handling processes were highly valued for protecting physicians from unfounded accusations while ensuring reasonable patient grievances were addressed. Multidepartmental collaboration and information-sharing mechanisms, along with third-party coordinator intervention (such as physician-patient relations offices, AI assistants, and patient companions), could effectively reduce information gaps and misunderstandings, ensuring continuity and smoothness in diagnostic and treatment processes. As a member of the physician-patient relations office said,

Patients always feel that they are the weaker party, but in fact they already have multiple channels for providing feedback. Healthcare workers are quite difficult because various parties may make all kinds of demands on them. Medical institutions should provide health care workers with more psychological, financial, and other forms of support, and ensure their safety, so that healthcare workers can focus on clinical work and form a virtuous cycle.

[A03]

#### Process Optimization and Patient Health Education

Physicians believed that streamlined processes could help shorten patient waiting times and reduce information delays. Additionally, providing patient health education to empower them to play an active role in their treatment was crucial for maintaining high-quality communication. In particular, patient education and health literacy improvement programs could help patients better understand medical information and actively participate in medical discussions, thereby reducing communication barriers caused by information asymmetry. As one physician explained,

If the appointment and check-in processes are straightforward and patients receive basic guidance beforehand, they will arrive less stressed and better prepared. We can use that time for what truly matters, such as collaboratively determining the best next steps.

[Dr11]

#### Educational Training and Policy Promotion

Participants emphasized that education and policy interventions were key to maintaining and strengthening these facilitating factors. Embedding scenario-based communication training throughout medical education and professional development (such as role-playing, standardized patient simulations, and video reviews) could significantly enhance physicians’ practical communication abilities. Mentorship and exemplary demonstrations helped continuously reinforce communication skills in clinical environments, forming positive transmission. At the policy level, through patient rights and responsibility education, health care worker protection legislation, and performance assessments incorporating communication quality indicators, effective communication could become an important measure of medical services, incentivizing physicians to continuously improve communication abilities and ensuring sincere exchanges in safe environments. A member of the physician-patient relations office explained,

Physician-patient communication is not just one-time learning, but requires reminders, feedback, and support at every stage of professional careers.

[A05]

#### AI-Assisted Baseline Knowledge

In contrast to the communication barriers posed by fragmented internet information (as displayed in Theme 1), some physicians hold positive attitudes toward AI-assisted diagnosis when used by patients with adequate health literacy. They viewed it as a superior alternative to traditional, unreliable information sources. Highlighting this nuanced perspective, one physician stated,

I generally welcome these learning-oriented patients; at least most AI information is accurate. I’d rather they consult AI than turn to Baidu platform or listen to neighbors.

[Dr08]

Simultaneously, some physicians emphasized that AI diagnosis required professional oversight to reduce risks and ensure patient benefits.

I maintain an overall positive attitude toward AI diagnosis. Relying solely on AI for diagnostic decisions carries risks, but these risks can be managed through professional oversight, ultimately benefiting patients at the patient level.

[Dr02]

This divergence illustrates that the digital information environment acts as a double-edged sword, where its impact heavily depends on the patient’s underlying health literacy and the physician’s guiding approach.

## Discussion

### Principal Findings

This qualitative study explored the barriers and facilitators of physician-patient communication in Chinese tertiary hospitals from dual perspectives of physicians and patient relations coordinators, and organized these factors into a multilevel framework covering patient, physician, and system levels. At the patient level, barriers included individual background differences, inadequate expression and limited health literacy, and psycho-emotional states with expectation misalignment; facilitators included effective expectation management, good health literacy and communication readiness, and trust in physicians with shared decision-making. At the physician level, barriers involved communication style deficiencies and empathy gaps as well as time pressure constraints; facilitators included active listening and patient-centeredness, empathy and emotional support, and clear explanations with cultural adaptability. At the system level, barriers encompassed hospital environment and medical visit settings, legal and policy deficiencies, insufficient communication training, and media-driven distrust with digitally mediated information challenges; facilitators included institutional support and security assurance, educational training and policy promotion, process optimization and patient health education, and AI-assisted baseline knowledge.

Consistent with the propositions of information ecology theory [28], our findings emphasize that physician-patient communication is not determined by individual factors alone but is embedded in contextual environments. This suggests that improving health care interactions cannot be achieved solely by enhancing physicians’ professional skills. Similar to previous research [29-31], we found that excessive administrative burdens and conflict management responsibilities undermine physicians’ ability to engage in effective communication with patients. Redistributing nonmedical responsibilities to appropriate administrative personnel or digital systems may alleviate physicians’ workload, reduce communication fatigue, and ultimately foster more meaningful, patient-centered interactions. Administrators from patient relations coordinators emphasized systemic solutions, including third-party mediation mechanisms, cross-departmental coordination, and a zero-tolerance policy for violent behavior. These measures are often overlooked but are important drivers for improving communication between physicians and patients. These perspectives challenge the dominant focus on individual-level communication training. However, it allows physicians to focus on their core medical responsibilities with peace of mind.

Furthermore, our findings highlight the need to attend to patient-level barriers and facilitators that influence physician-patient communication. As proposed by Inui and Carter [32], physician-patient communication is a reciprocal exchange that demands active participation from both parties. While patient-centered communication requires balanced information exchange and complementary roles, the communication gap often extends beyond simple information asymmetry to include disparities in health literacy, shared understanding, and collaborative care capacity [33,34]. To bridge this gap, comprehensive patient education programs are essential and should address 2 key dimensions. First, education must narrow the knowledge divide between medical professionals and the general public. This entails moving beyond traditional paternalistic models toward a collaborative framework in which patients become informed partners, equipped with basic health literacy, a clear understanding of treatment options, and realistic expectations regarding therapeutic outcomes [35]. Second, such programs should build communication competencies, empowering patients to make the most of limited consultation time by articulating symptoms clearly, asking relevant questions, expressing concerns appropriately, and participating in shared decision-making [36]. When patients proactively express their expectations and concerns, physician-patient communication becomes more efficient and meaningful. This, in turn, fosters mutual tolerance, understanding, and respect, thereby promoting a supportive and reciprocal communicative dynamic [37].

Within a multilevel framework, AI functions as a digitally mediated environmental factor that introduces new challenges to physician-patient communication. Although existing studies have often compared patient preferences for AI- versus physician-generated communication [38], or evaluated the relative quality of AI-assisted and clinician communication [39], less is known about how physicians respond when patients bring AI-generated information into real-world consultations. Our findings address this gap by showing that physicians did not view AI-informed communication as uniformly beneficial or harmful, but evaluated it according to how patients used, interpreted, and communicated AI-generated information during clinical encounters. Consistent with recent scoping review evidence [39], our findings suggest that AI-informed communication was not perceived as uniformly harmful or beneficial. On the one hand, AI may improve patients’ baseline knowledge and support more informed questioning, thereby creating conditions for more patient-centered communication. On the other hand, it may also introduce new communication pressures when AI-generated information is inaccurate, decontextualized, or treated by patients as a substitute for clinical judgment. Importantly, physicians in our study did not uniformly reject AI-informed patients. Rather, they distinguished between patients who used AI as a learning tool to better understand their condition and those who used AI-generated advice to challenge professional judgment. This finding suggests that the impact of AI on physician-patient communication depends less on the presence of AI itself than on how AI-generated information is interpreted, communicated, and managed within clinical encounters.

### Strengths and Limitations

Grounded in the context of Chinese tertiary hospitals, this study breaks the limitation of single-stakeholder views by integrating the experiences of practicing physicians with the insights of patient relations coordinators. It bridges micro-level clinical encounter practices with macro-level organizational processes and further identifies the double-edged sword effect of digital transformation on physician-patient communication. Collectively, this work provides constructive reflections for optimizing physician-patient interactions in high-workload clinical environments.

Several limitations should be noted. First, this study focused on tertiary hospitals in Guangzhou and Shenzhen, 2 economically developed cities in southern China. While these settings provide valuable insights into high-complexity health care environments, findings may not fully reflect communication dynamics in different hospital levels or geographic regions. Second, the qualitative design offers rich contextual understanding but cannot quantify the relative importance of identified factors or establish causal relationships. Third, the study was conducted during a specific timeframe and may not capture evolving digital health trends. Future research should examine communication patterns across diverse health care settings and incorporate mixed methods approaches to validate these findings and assess intervention effectiveness.

### Conclusion

This qualitative study of frontline physicians and patient relations coordinators within tertiary hospitals in China identified the key barriers and facilitators to physician-patient communication. Our findings support that effective physician-patient communication is influenced by a variety of factors, including patient-, physician-, and system-level factors. In the modern era, physician-patient communication is challenged by consumerist medical attitudes, the internet age, and societal changes, but also benefits from new mechanisms such as third-party coordination and technological assistance. To achieve effective communication, it is necessary to understand these complex factors and establish a relationship based on mutual understanding and respect, while also implementing systematic education and training, policy support, and organizational improvements to optimize the communication environment.

## Supplementary material

10.2196/87947 Multimedia Appendix 1 Interview guide.

## Abbreviations

AI: artificial intelligence
COREQ: Consolidated Criteria for Reporting Qualitative Research
