# Supplementary material for: Barriers and Facilitators to Physician-Patient Communication in Chinese Tertiary Hospitals From the Perspectives of Hospital-Based Physicians and Patient Relations Coordinators: Qualitative Study
Source: Interact J Med Res. 2026 Jun 11;15:e87947. doi: 10.2196/87947 (PMC13255652; doi:10.2196/87947)
Supplement: Multimedia Appendix 1 [file ijmr-v15-e87947-s001.docx]

**Interview Guide**

Thank you very much for taking time out of your busy schedule to participate in our patient-physician communication research project. Your insights and experiences are invaluable to our understanding of the current state of patient-physician communication and potential areas for improvement. The interview will last approximately 1 hour and will primarily take the form of a question-and-answer session. Interview content will be used exclusively for the research project analysis, and we guarantee strict confidentiality of your privacy and interests. We sincerely appreciate your support and cooperation. The interview outline is provided below:

1.In your interactions with patients, what needs or expectations do you feel they generally have?

2.In your view, what kind of patients would you consider “good patients” or “easy-to-communicate-with patients”?

3.In your view, what kind of doctors would you consider “good doctors” or “good communicators”?

4.In your opinion, what should doctors do, what should patients do, and what external conditions (e.g., platform, policies, daily work arrangements) are needed to achieve a high-quality doctor–patient communication?

5.What factors (such as barriers or facilitators) do you think most influence the quality of doctor–patient communication? What are your views or suggestions for improving doctor–patient communication quality?
